# Monitoring Ca^2+^ elevations in individual astrocytes upon local release of amyloid beta in acute brain slices

**DOI:** 10.1016/j.brainresbull.2016.12.007

**Published:** 2018-01

**Authors:** Olga Tyurikova, Kaiyu Zheng, Annika Rings, Anna Drews, David Klenerman, Dmitri A. Rusakov

**Affiliations:** a UCL Institute of Neurology, University College London, Queen Square, London WC1 3BG, UK; b Institute of Neuroscience, University of Nizhny Novgorod, 603950 Nizhny Novgorod, Russia; c Department of Chemistry, University of Cambridge, Lensfield Road, Cambridge, UK

**Keywords:** Amyloid beta, Astroglia, Hippocampus, Calcium imaging, Fluorescence lifetime imaging

## Abstract

The pathogenesis of Alzheimer’s disease (AD) is thought to involve acute neurotoxic effects exerted by oligomeric forms of amyloid-β 1-42 (Aβ). Application of Aβ oligomers in physiological concentrations have been shown to transiently elevate internal Ca^2+^ in cultured astroglia. While the cellular machinery involved has been extensively explored, to what degree this important signalling cascade occurs in organised brain tissue has remained unclear. Here we adapted two-photon excitation microscopy and calibrated time-resolved imaging (FLIM), coupled with patch-clamp electrophysiology, to monitor Ca^2+^ concentration ([Ca^2+^]) inside individual astrocytes and principal neurons in acute brain slices. Inside the slice tissue local micro-ejection of Aβ in sub-micromolar concentrations triggered prominent [Ca^2+^] elevations in an adjacent astrocyte translated as an approximately two-fold increase (averaged over ∼5 min) in basal [Ca^2+^]. This elevation did not spread to neighbouring cells and appeared comparable in amplitude with commonly documented spontaneous [Ca^2+^] rises in astroglia. Principal nerve cells (pyramidal neurons) also showed Ca^2+^ sensitivity, albeit to a lesser degree. These observations shed light on the extent and dynamics of the acute physiological effects of Aβ on brain cells in situ, in the context of AD.

## Highlights

• Local application of Aβ oligomers raises astroglial Ca^2+^ in acute brain slices.
• Ca^2+^ elevations do not spread to neighbouring astroglia.
• Principal neurons are less sensitive to Aβ oligomer application.

## 1 Introduction

Extracellular plaques containing insoluble conjugates of amyloid beta1-42 (Aβ 1-42, the amyloid precursor protein (APP) cleavage product) are a classical indicator of Alzheimer’s disease (AD). Although Aβ has been shown to dysregulate synaptic proteins triggering degeneration of dendritic spines (Spires-Jones and Hyman, 2014), the underlying cellular machinery is poorly understood. In this context, acute application of Aβ to brain cells *in situ* has been an important tool to discern its molecular targets and the metabolic cascades involved in the cellular response (Jo et al., 2011, Wang et al., 2004). It has been previously shown that the Aβ oligomers taken from the cerebrospinal fluid (CSF) of Alzheimer’s patients impair synaptic plasticity in acute brain slices, the effect preventable by the addition of Aβ antibodies (Walsh et al., 2002). It appears that Aβ targets, directly or indirectly, metabotropic glutamate receptors and the prion protein receptor in the synaptic environment, inducing synaptic dysfunction and eventually cell death (Chen et al., 2010, Lauren et al., 2009, Um et al., 2013). Furthermore, once inside principal neurons, Aβ rapidly enhance excitatory synaptic transmission, likely because of the insertion of Ca^2+^-permeable AMPA receptors (Whitcomb et al., 2015).

Whilst the evidence for direct influence of Aβ on nerve cells is rapidly accumulating, little is known about its effects on the other omnipresent brain cell type, astroglia. The key role of astroglia in providing powerful glutamate uptake and extracellular potassium buffering and thus protecting nerve cells against runaway neurotoxicity has long been acknowledged (Vernadakis, 1996). Astrocytes attract intense attention because they have recently emerged as important players in regulating the activity of local synaptic circuitry (reviewed in ((Araque et al., 2014, Bezzi et al., 2001, Haydon and Carmignoto, 2006, Rusakov et al., 2014, Rusakov et al., 2011, Volterra and Meldolesi, 2005)). Being electrically unexcitable cells, astroglia appear to employ prominent Ca^2+^ elevations and regenerative Ca^2+^ waves to integrate and propagate physiological signals (recently reviewed in (Bazargani and Attwell, 2016, Rusakov, 2015, Volterra et al., 2014)). Intriguingly, the key signalling cascades classically associated with neurotoxicity involve excessive Ca^2+^ entry in brain cells (Witt et al., 1994), and recent work has documented prominently increased Ca^2+^ activity in both neurons and astroglia near Aβ plaques in the AD mice model *in vivo* (Busche et al., 2008, Kuchibhotla et al., 2009).

Intracellular Ca^2+^ elevations induced in cultured astroglia by the acute application of Aβ oligomers have long been documented (Abramov et al., 2003, Demuro et al., 2005). Such observations have prompted several important mechanistic hypotheses pertinent to the underlying cellular cascades (Abeti et al., 2011, Abramov et al., 2011, Demuro et al., 2011, Verkhratsky and Parpura, 2010). However, earlier experimental studies usually employed bath application of monomer and oligomers to cell cultures at relatively high concentrations, even though the physiological level of oligomers is likely to be in the picomolar range. In this context, we recently reported that local application of purified Aβ1-42 oligomers at physiological concentrations triggered transient Ca^2+^ elevations in astroglia and, to a lesser degree, in neurons (Drews et al., 2016). However, whether observations in cultured astroglia – which appears pancake-shaped and has no surrounding neuropil, extracellular milieu or gap-junction connected neighbours – can be directly extrapolated to sponge-like astrocytes *in situ* is a subject of debate (Araque et al., 2014, Bazargani and Attwell, 2016, Hamilton and Attwell, 2010, Volterra and Meldolesi, 2005). In addition, free diffusion in organised brain tissue could be highly restricted thus potentially limiting the spatial extent and the magnitude of their effects.

We therefore sought to test whether Aβ1-42 exerts any detectable influence on astroglial and neuronal Ca^2+^ in the neuropil of acute brain slices using two-photon excitation microscopy. Because fluorescence intensity –dependent Ca^2+^ monitoring in astrocytes *in situ* could be biased by concomitant fluctuations in the cell cytosolic volume, focus plane, or tissue scattering (Rusakov, 2015) we also used a recently developed fluorescence lifetime imaging (FLIM) technique which overcomes such uncertainties (Zheng et al., 2015).

## 2 Materials and methods

### 2.1 Aβ42 aggregation

A Biosep SEC-s2000 size exclusion column (Phenomenex) was employed to purify HiLyte Fluor 647 Aβ 42 (Cambridge Bioscience LDT), with pH 7.4 SSPE (0.01 M Na2HPO4, 0.15 M NaCl, 1 mM EDTA) as the running buffer. Prior to purification, the peptide was kept in ice, flash frozen immediately after purification and stored at −80 °C. In experimental aliquots, purified Aβ 42 was diluted to 500 nM in PBS and left shaking at 37 °C, 200 rpm for 5 h. It was centrifuged at 14,500*g* for 10 min and then diluted to the required concentration in L15 medium.

### 2.2 Hippocampal slice preparation

Acute hippocampal slices (350 μm) thick were prepared from P21–24 Sprague-Dawley rats, in full compliance with the national guidelines, the European Communities Council Directive of 24 November 1986, and the European Directive 2010/63/EU on the Protection of Animals used for Scientific Purposes. Slices were prepared in an ice-cold slicing solution containing (in mM) NaCl 87, sucrose 75, NaHCO_3_ 25, KCl 2.5, NaH_2_PO_4_ 1.25, MgCl_2_ 7, CaCl_2_ 0.5, glucose 25 (osmolarity 300–305 mOsM) stored in the slicing solution at 34 °C for 20 min before transferred for 40 min storage in solution containing (in mM) NaCl 119, NaHCO_3_ 25, KCl 2.5, NaH_2_PO_4_ 1.25, MgSO_4_ 1.3,CaCl_2_ 2.5, and glucose 11 (osmolarity 300–305 mOsM). All solutions were continuously bubbled with 95% O_2_/5% CO_2._

### 2.3 Electrophysiology and dye loading

Whole-cell patch-clamp recordings of electrically-passive *stratum radiatum* astrocytes and CA1 pyramidal neurons were performed. During recordings slices were maintained in solution containing (in mM) NaCl 119, NaHCO_3_ 25, KCl 2.5, NaH_2_PO_4_ 1.25, MgSO_4_ 1.3,CaCl_2_ 2.5, and glucose 11 (osmolarity 300–305 mOsM). Whole-cell recordings were obtained with patch pipettes (3–4 MΩ) with an intracellular solution containing (in mM): KCH_3_O_3_S 135, HEPES 10, disodium phosphocreatine 10, MgCl_2_ 4, Na_2_ATP 4, NaGTP 0.4 (pH adjusted to 7.2 with KOH, osmolarity 290–295 mOsM). Protoplasmic astrocytes were identified by small soma size, low input resistance (<10 MΩ), linear current-voltage relationship and low rest membrane potential (<–80 mV). Pyramidal neurons were held at −70 mV. The cell impermeable Ca^2+^ indicator OGB-1 (200 μM) was added to the internal solution. In order visualise gap- junction coupled (GJC) astrocytes at least 20 min was required for dye diffusion through the syncytium. Aβ 42 was applied via puff pipette (3–4 MΩ) next to one of the GJC astrocytes or CA1 neuron and Ca^2+^ was monitored in this and neighbouring cells.

### 2.4 Two-photon excitation imaging and FLIM

Two-photon excitation by femtosecond infrared laser pulses was used to restrict excitation and emission collection to a thin focal excitation plane 50–110 μm deep into the slice. The imaging system was based on the Femto2D microscope equipped with a Becker and Hickl FLIM detector (Femtonics, Budapest). We ensured that no contaminating fluorescence signal was collected from damaged tissue near the slice surface, and no autofluorescence was detected at these depths (before applying OGB-1). A short-pass 700 nm filter was placed in front of the detector to block out any escaped light from the laser source. A bandpass 500–560 nm filter was also placed in front of the detector to spectrally separate OGB-1 signal from reference fluorescence of SR101 (see below) and Alexa Fluor 594. The two-photon laser source was a Newport-Spectraphysics Ti:Sapphire MaiTai laser pulsing at 80 Mhz, with a pulse width of ∼220 fs and a wavelength of 800 nm optimized for OGB-1 excitation. The laser power was kept below 8 mW under the objective at all times for slice preparations, to minimize phototoxic damage. In fluorescence intensity measurements, experiment-wise baseline trends due to photobleaching, AM dye extrusion or focus drift (normally <20% deviation from the original baseline) were subtracted using linear regression.

In fluorescence lifetime imaging (FLIM) experiments, images were acquired at a laser line scanning rate of 500 lines per second and stored as a 256 × 512 × 512 x n (*t*,*x,y,T*) data cube representing an *x-y* images with distribution of nanosecond delay time (*t*) of photons at each pixel over the frame duration (*T*). Average image acquisition times were 120–300 s (maximum laser exposure time of <100 s to minimize phototoxic damage) depending on the total photon count, and the maximum photon count rate was on average <10^5^ s^−1^ which is well below effect of photon pile-up (maximal photon count of the system was near 10^8^ s^−1^). In FLIM tests, the fluorescence intensity were routinely recovered from FLIM data by integration of (non-normalised) photon counting data at any given pixel or ROI.

### 2.5 OGB-1 calibration for [Ca^2+^] readout

The calibration protocol was similar to the standard calibration method provided by the Invitrogen Ca^2+^ calibration buffer kit manual, and was further refined as described earlier (Zheng et al., 2015). In brief, to match Ca^2+^ buffering dynamics to that of OGB-1 more closely, the standard 10 mM chelating agent EGTA was replaced with 10 mM BAPTA, and the solution constituents were replaced with our experimental intracellular solution (see below). pH was adjusted using KOH, and the KCl concentration in the intracellular solution was adjusted accordingly, to keep ion constituents in the solution unchanged. The estimated [Ca^2+^] was finely adjusted using WEBMAXC program at Stanford by Chris Patton (http://www.stanford.edu/∼cpatton/webmaxcS.htm).

## 3 Results

### 3.1 Monitoring the effects of Aβ oligomers in astroglia bulk loaded with Ca^2+^ indicator

**Fig. 1:**
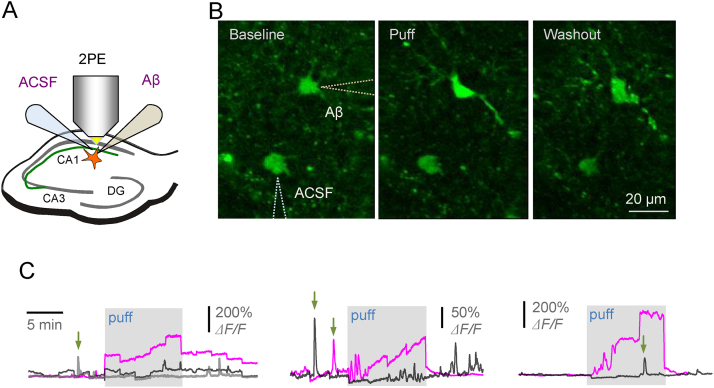
Local application of Aβ oligomers triggers increases in [Ca^2+^]-dependent fluorescence in the astroglia bulk loaded with OGB-1 AM in acute brain slices. (A) Diagram depicting experimental arrangement; hippocampal slices areas, two-photon excitation imaging, and two pressurised micropipettes are indicated. (B) Example of individual astrocytes imaged (OGB-1 intensity channel) in baseline conditions, during the 10 min local Aβ oligomer puff, and during washout, as indicated; pipette positions are depicted (dotted lines). (C) Three characteristic examples depicting increases in [Ca^2+^] fluorescence (OGB-1) upon application of Aβ oligomer (blue traces), with no detectable effects during ACSF application in the same experiment (black traces; grey trace in the left graph depicts a no-puff example); arrows, examples of spontaneous [Ca^2+^] elevations in astroglia; staggered trace shape reflect periodic focus corrections during long recording sessions.

To enable intracellular Ca^2+^ monitoring under acute application of Aβ to astroglia *in situ*, we incubated acute hippocampal slices with a high-affinity Ca^2+^ indicator Oregon Green BAPTA-1 (cell-permeable OGB-1 AM, at 5 μM; Materials and Methods). In some experiments, we also used sulfarhodamine101 (SR101, 10 μM), a widely used empirical marker of astroglia, to confirm positions of astrocyte bodies, as detailed previously (Zheng et al., 2015); both indicators could be excited in two photon mode at λ = 800 nm. We thus set out to image individual astroglia in hippocampal area CA1 while monitoring Ca^2+^ dependent fluorescence in the chromatically separated OGB-1 channel (Fig. 1A). In control experiments, we could routinely induce Ca^2+^ rises in monitored astroglia by bulk application of the non-specific group I mGluR agonist DHPG, this confirming cell viability. Subsequently, because Aβ application produced a detectable effect, the DHPG positive-control tests were omitted.

Because freely diffusing Aβ is likely to aggregate or get absorbed, or both, by cellular structures filling the brain tissue volume, we arranged local Aβ application through a pressurized micro-pipette near the cell of interest (Fig. 1A–B). This also enabled us to measure the local rather than bulk effects of Aβ. Similar to our earlier experiments, the pressure puff efflux was controlled by monitoring the tip-ejected stream of Alexa indicator which was added to the pipette medium (Scott et al., 2008). However, pressure puffs induce local mechanical disturbance, and it has been known that astrocyte Ca^2+^ could be sensitive to such disturbances (Angulo et al., 2004, Newman, 2001). To control for these concomitants we therefore placed another pressurised pipette, filled with bath solution (no Aβ), in the nearby area (Fig. 1A–B).

We found that a 10 min local puff of aggregated Aβ (0.5 μM total monomer in the pipette solution, diluted 10–20-fold as it reaches the target cell) induced a prominent Ca^2+^ elevation which subsided after the end of the puff (Fig. 1B–C). A similar puff with the control solution applied nearby (bath medium) in the same experiment had no effect on astroglial Ca^2+^, thus arguing against the mechanical artefacts involved (Fig. 1C; here, because of large fluorescence signal variability in these tests we documented individual experiments rather than a statistical summary). Nonetheless, slow changes of Ca^2+^-dependent fluorescence intensity associated with Aβ oligomer application in these experiments might still reflect changes in the cytosolic volume with the indicator or in the optical properties (scattering and absorption) of local slice tissue (Rusakov, 2015). To avoid such concomitants we turned to the FLIM-based Ca^2+^ concentration ([Ca^2+^]) monitoring method that takes advantage of the fact that OGB-1 fluorescence lifetime is highly sensitive to free nanomolar Ca^2+^ (Wilms and Eilers, 2007, Wilms et al., 2006, Zheng et al., 2015). While providing direct [Ca^2+^] readout, this FLIM measure does not depend on the dye concentration, the multiple concomitants of cell function, or the optical properties of organised brain tissue (Zheng et al., 2015).

### 3.2 FLIM-based measurements of basal astroglial Ca^2+^ with OGB-1 loaded through gap-junction connected cells

**Fig. 2:**
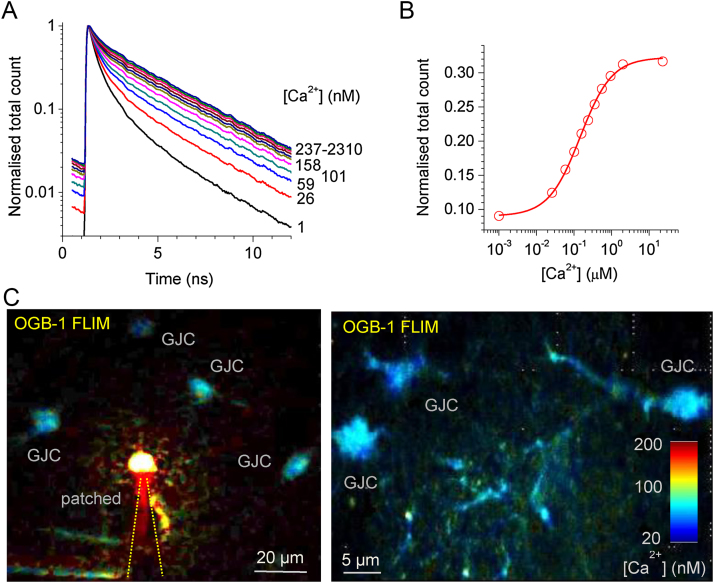
Time-resolved (two-photon excitation FLIM-based) monitoring of intracellular [Ca^2+^] in unperturbed astroglia in situ. (A) Fluorescence life time of OGB-1 in calibrated solutions of clamped [Ca^2+^] (concentrations are indicated in nM). (B) Summary calibration curve (normalised total photon count, see (Zheng et al., 2015) for detail). (C) Examples of FLIM-enabled [Ca^2+^] mapping in patched (left) and gap-junction-connected (GJC) astroglia in acute hippocampal slices; false colour concentration scale bar applies throughout.

First, we set to calibrate the FLIM system used in the present experiments (Femtonics, Budapest), for the [Ca^2+^] readout, as detailed previously for an alternative imaging system (Zheng et al., 2015). The nanosecond timescale fluorescence decay of OGB-1 showed prominent sensitivity to [Ca^2+^] between 1 and 100 nM, fully in line with previous observations (Wilms and Eilers, 2007, Wilms et al., 2006, Zheng et al., 2015) (Fig. 2A). Correspondingly, the normalised total photon count provided the calibration relationship (Fig. 2B) which was required to map, pixel-by-pixel, intracellular [Ca^2+^] across the imaged samples.

We next held individual astroglia in whole cell mode filling them with 200 μM OGB-1: because individual astrocytes are interconnected with their neighbours via multiple gap junctions, the fluorescent indicator diffuses into multiple gap-junction connected (GJC) cells (Fig. 2C). Importantly, these GJC cells remain virtually unperturbed by the pipette dialysis (Zheng et al., 2015) and the FLIM-based [Ca^2+^] readout does not depend on the OGB-1 in these cells, thus providing an opportunity to monitor astroglial [Ca^2+^] in near-physiological conditions. We therefore focused on GJC cells in our measurements.

This calibration relationship (Fig. 2B) was thus employed to map [Ca^2+^] in the focal plane of view (two-photon excitation optical layer) over the bodies of GJC astroglia using a false colour table for illustration purposes (Fig. 2C). At the same time, [Ca^2+^] values were decoded and documented using raw FLIM data for all pixels throughout.

### 3.3 Aβ oligomer application elevates basal [Ca^2+^] in the immediately adjacent but not distant astroglia in situ

**Fig. 3:**
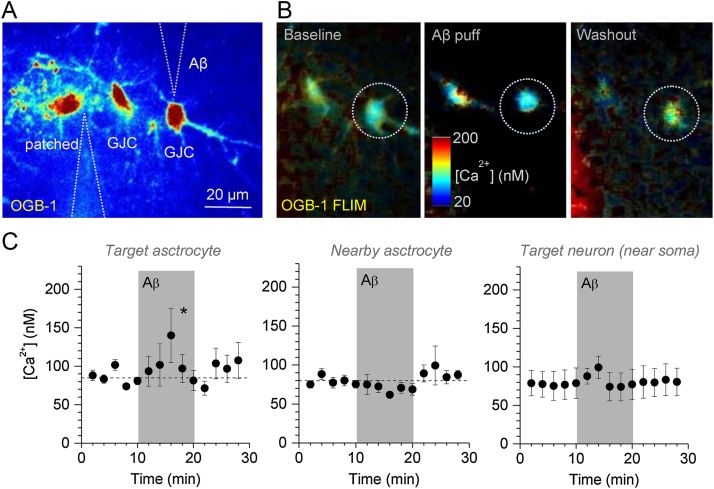
Local Aβ oligomer application triggers reversible [Ca^2+^] elevations in the adjacent astroglia. (A) Example of the experimental arrangement, with three astroglia (one patched and two GJC), patch pipette, and the Aβ oligomer application pipette indicated. False colour intensity image acquired in OGB-1 channel. (B) Monitoring astroglial [Ca^2+^] in the experiment depicted in (A): OGB-1 FLIM based [Ca^2+^] maps in baseline conditions, during the Aβ oligomer application, and during washout, as indicated; dotted circle, target astrocyte. (C) Statistical summary (mean ± SEM) of experiments depicted in (A-B), including the immediately adjacent astroglia (left, n = 4), neighbouring astrocytes (centre, n = 10), and CA1 pyramidal neurons (recorded near soma due to low photon count in thin dendrites, n = 3); * p = 0.023 (because [Ca^2+^] peaked at different times post-application in individual cells, the comparison was made in each cell over a 10 min [Ca^2+^] peak interval against a 5 min baseline interval preceding the application; paired-sample *t*-test).

We therefore used the FLIM-based method to monitor [Ca^2+^] in individual GJC cells visualised 50–100 μm from the patched astrocyte. In each experiment, the Aβ oligomer −filled pressure micro-pipette tip was advanced to the 2–3 μm proximity of the selected cell body (Fig. 3A). After a ∼10 min baseline [Ca^2+^] monitoring, Aβ oligomers were applied for 10 min. The tests showed that the application of Aβ oligomers induced a transient elevation of basal astroglial [Ca^2+^], on average from 70 to 80 nM to 140–150 nM (Fig. 3B–C, left). These results were qualitatively consistent with our observations using fluorescence intensity measures (Fig. 1) while ruling out the concomitant effects pertinent to the possible changes in cell volume, focus or light scattering that might have been present in fluorescence intensity measures.

Interestingly, neighbouring GJC astroglia (20–50 μm from the tested cell) showed no Ca^2+^ sensitivity to Aβ oligomer application in these experiments (Fig. 3C, centre). This suggests that Aβ oligomers may not be able to travel far in organised brain tissue and that astroglial [Ca^2+^] do not readily propagate to neighbouring cells via gap junctions in this case. A similar protocol applied to monitor [Ca^2+^] in principal neurons in the same hippocampal area (CA1 pyramidal cells) showed, if anything, less significant Aβ-induced [Ca^2+^] elevations (Fig. 3C, right). However, FLIM-based observations in neurons were complicated by the relatively low photon count, hence low signal-to-noise ratios, in thin dendrites, in particular conditions of the Aβ application protocol. Thus, the imaging was carried out in ROIs close to the neuronal soma: detecting effects of Aβ on a smaller scale or in thin neuronal dendrites would therefore require a special investigation.

## 4 Discussion

### 4.1 Importance of in situ observations pertinent to Aβ effects in brain cells

The overall aim of the present study was to ascertain whether the established Ca^2+^ sensitivity of astroglia to the physiological concentrations of Aβ oligomers in primary cell culture is relevant to astrocytes *in situ*. The rationale for this investigation was three-fold.

Firstly, extrapolating experimental observations obtained in cultured astroglia to astrocytes in organised brain tissue has been a much debated task. This is mainly because of dramatic differences in morphology and function between the two. Indeed, cultured astroglia has a flattened, pancake-like shape, sometimes covering large areas between neurons. In contrast, the bulk of astroglial morphology *in situ* has a sponge-like, space-filling architecture, contributing significantly to the 3D microenvironment of synaptic connections between neurons. A similarly prominent disparity has been reported regarding astroglial physiology, in particular the expression and function of signalling molecules including receptors, transporters and cellular devices such as mitochondria or Ca^2+^ stores (reviewed in (Araque et al., 2014, Di Castro et al., 2011, Hamilton and Attwell, 2010, Haydon and Carmignoto, 2006, Parpura and Verkhratsky, 2012, Verkhratsky and Kettenmann, 1996, Vernadakis, 1996, Volterra and Meldolesi, 2005)).

Secondly, astroglia or neurons in culture do not face the narrow extracellular space which, in addition to providing a natural obstacle to diffusion in organised brain tissue, also contains a milieu of extracellular matrix proteins and other large macromolecules. Thus, our earlier experiments in culture, while being important for establishing the dose-response characteristics of Aβ application (Drews et al., 2016), are unlikely to represent conditions of the diffuse Aβ spread which could be relevant to *in vivo* models of AD (Busche et al., 2008, Kuchibhotla et al., 2009).

Finally, cell-permeable indicators, such as OGB-1 AM, could, at least in some conditions, be taken up and hydrolysed inside intracellular organelles, such as ER or mitochondria, rather than being contained entirely within the cell cytoplasm. In that case, it might be difficult to unambiguously the origin of Ca^2+^-dependent fluorescence changes.

### 4.2 Concluding remarks

In the present work, we used a recently developed experimental approach in which intracellular [Ca^2+^] is monitored in astroglia that are filled with a soluble Ca^2+^ indicator through gap junctions using a FLIM technique (Zheng et al., 2015). This method ensured that the imaged cells were virtually unperturbed by dialysis while retaining the indicator inside the cytoplasm. More importantly, once calibrated, the FLIM-based technique provided direct [Ca^2+^] readout which was independent of all major concomitants of cell function (Zheng et al., 2015). We thus found that Aβ oligomers did induce significant [Ca^2+^] elevations in individual astroglia, while having lesser effects in neurons. This confirms our earlier observations in culture (Drews et al., 2016). In our present experiments Aβ oligomers were applied in relatively high local concentrations compared to the physiological levels (∼1 nM near cell surface versus 1 pM in cerebrospinal fluid), but it had no detectable effects on neighbouring cells. This observation suggests that the extracellular milieu in organised brain tissue does represent a substantial obstacle to the diffuse spread of Aβ. It also argues that Aβ-induced astroglial [Ca^2+^] rises do not readily propagate via gap junctions to other astroglia.

The present observations thus improve our understanding of direct physiological actions of Aβ oligomers on astroglia *in situ*, also providing novel insights into the efficiency and spread of such actions in organised brain tissue. The Ca^2+^ monitoring imaging method we adapted and tested should help future studies aiming to probe Aβ action directly in brain slices or *in vivo*. Here we were able to detect an acute, albeit short-lived, effect of Aβ on astrocyte Ca^2+^ *in situ*. The transient nature of the effect was likely due to the adopted experimental design in which application of Aβ was also transient. To establish the extent and the endogenous dynamics of such effects *in vivo*, in conditions compatible with AD, is an important question which requires a separate study.

## Conflict of interest

The authors declare no potential conflicts of interest with respect to the authorship and/or publication of this article.

## Author contribution

O. Tyurikova carried out experimental studies; K. Zheng designed FLIM calibration experiments and performed FLIM analyses; A. Rings did control DHPG and Aβ tests in astroglia; A. Drews obtained Aβ oligomers; D. Rusakov and D. Klenerman narrated the study; D. Rusakov wrote the manuscript which was subsequently modified by all authors.

## References

[bib0005] Abeti R., Abramov A.Y., Duchen M.R. (2011). beta-amyloid activates PARP causing astrocytic metabolic failure and neuronal death. Brain.

[bib0010] Abramov A.Y., Canevari L., Duchen M.R. (2003). Changes in intracellular calcium and glutathione in astrocytes as the primary mechanism of amyloid neurotoxicity. J. Neurosci..

[bib0015] Abramov A.Y., Ionov M., Pavlov E., Duchen M.R. (2011). Membrane cholesterol content plays a key role in the neurotoxicity of beta-amyloid: implications for Alzheimer's disease. Aging Cell.

[bib0020] Angulo M.C., Kozlov A.S., Charpak S., Audinat E. (2004). Glutamate released from glial cells synchronizes neuronal activity in the hippocampus. J. Neurosci..

[bib0025] Araque A., Carmignoto G., Haydon P.G., Oliet S.H., Robitaille R., Volterra A. (2014). Gliotransmitters travel in time and space. Neuron.

[bib0030] Bazargani N., Attwell D. (2016). Astrocyte calcium signaling: the third wave. Nat. Neurosci..

[bib0035] Bezzi P., Domercq M., Vesce S., Volterra A. (2001). Neuron-astrocyte cross-talk during synaptic transmission: physiological and neuropathological implications. Prog. Brain Res..

[bib0040] Busche M.A., Eichhoff G., Adelsberger H., Abramowski D., Wiederhold K.H., Haass C., Staufenbiel M., Konnerth A., Garaschuk O. (2008). Clusters of hyperactive neurons near amyloid plaques in a mouse model of Alzheimer's disease. Science.

[bib0045] Chen S.G., Yadav S.P., Surewicz W.K. (2010). Interaction between human prion protein and amyloid-beta (A beta) oligomers role of N-terminal residues. J. Biol. Chem..

[bib0050] Demuro A., Mina E., Kayed R., Milton S.C., Parker I., Glabe C.G. (2005). Calcium dysregulation and membrane disruption as a ubiquitous neurotoxic mechanism of soluble amyloid oligomers. J. Biol. Chem..

[bib0055] Demuro A., Smith M., Parker I. (2011). Single-channel Ca2+ imaging implicates A beta 1–42 amyloid pores in Alzheimer's disease pathology. J. Cell Biol..

[bib0060] Di Castro M.A., Chuquet J., Liaudet N., Bhaukaurally K., Santello M., Bouvier D., Tiret P., Volterra A. (2011). Local Ca2+ detection and modulation of synaptic release by astrocytes. Nat. Neurosci..

[bib0065] Drews A., Flint J., Shivji N., Jonsson P., Wirthensohn D., De Genst E., Vincke C., Muyldermans S., Dobson C., Klenerman D. (2016). Individual aggregates of amyloid beta induce temporary calcium influx through the cell membrane of neuronal cells. Sci. Rep..

[bib0070] Hamilton N.B., Attwell D. (2010). Do astrocytes really exocytose neurotransmitters?. Nat. Rev. Neurosci..

[bib0075] Haydon P.G., Carmignoto G. (2006). Astrocyte control of synaptic transmission and neurovascular coupling. Physiol. Rev..

[bib0080] Jo J., Whitcomb D.J., Olsen K.M., Kerrigan T.L., Lo S.C., Bru-Mercier G., Dickinson B., Scullion S., Sheng M.G., Collingridge G., Cho K. (2011). A beta(1–42) inhibition of LTP is mediated by a signaling pathway involving caspase-3, Akt1 and GSK-3 beta. Nat. Neurosci..

[bib0085] Kuchibhotla K.V., Lattarulo C.R., Hyman B.T., Bacskai B.J. (2009). Synchronous hyperactivity and intercellular calcium waves in astrocytes in Alzheimer mice. Science.

[bib0090] Lauren J., Gimbel D.A., Nygaard H.B., Gilbert J.W., Strittmatter S.M. (2009). Cellular prion protein mediates impairment of synaptic plasticity by amyloid-beta oligomers. Nature.

[bib0095] Newman E.A. (2001). Propagation of intercellular calcium waves in retinal astrocytes and Muller cells. J. Neurosci..

[bib0100] Parpura V., Verkhratsky A. (2012). Homeostatic function of astrocytes: ca2+ and Na+ signalling. Transl. Neurosci..

[bib0105] Rusakov D.A., Zheng K., Henneberger C. (2011). Astrocytes as regulators of synaptic function: a quest for the Ca^2+^ master key. Neuroscientist.

[bib0110] Rusakov D.A., Bard L., Stewart M.G., Henneberger C. (2014). Diversity of astroglial functions alludes to subcellular specialisation. Trends Neurosci..

[bib0115] Rusakov D.A. (2015). Disentangling calcium-driven astrocyte physiology. Nat. Rev. Neurosci..

[bib0120] Scott R., Lalic T., Kullmann D.M., Capogna M., Rusakov D.A. (2008). Target-cell specificity of kainate autoreceptor and Ca^2+^-store-dependent short-term plasticity at hippocampal mossy fiber synapses. J. Neurosci..

[bib0125] Spires-Jones T.L., Hyman B.T. (2014). The intersection of amyloid beta and tau at synapses in Alzheimer's disease. Neuron.

[bib0130] Um J.W., Kaufman A.C., Kostylev M., Heiss J.K., Stagi M., Takahashi H., Kerrisk M.E., Vortmeyer A., Wisniewski T., Koleske A.J. (2013). Metabotropic glutamate receptor 5 is a coreceptor for alzheimer a beta oligomer bound to cellular prion protein. Neuron.

[bib0135] Verkhratsky A., Kettenmann H. (1996). Calcium signalling in glial cells. Trends Neurosci..

[bib0140] Verkhratsky A., Parpura V. (2010). Recent advances in (patho)physiology of astroglia. Acta Pharmacol. Sin..

[bib0145] Vernadakis A. (1996). Glia-neuron intercommunications and synaptic plasticity. Prog. Neurobiol..

[bib0150] Volterra A., Meldolesi J. (2005). Astrocytes, from brain glue to communication elements: the revolution continues. Nat. Rev. Neurosci..

[bib0155] Volterra A., Liaudet N., Savtchouk I. (2014). Astrocyte Ca^2+^ signalling: an unexpected complexity. Nat. Rev. Neurosci..

[bib0160] Walsh D.M., Klyubin I., Fadeeva J.V., Cullen W.K., Anwyl R., Wolfe M.S., Rowan M.J., Selkoe D.J. (2002). Naturally secreted oligomers of amyloid beta protein potently inhibit hippocampal long-term potentiation in vivo. Nature.

[bib0165] Wang Q.W., Walsh D.M., Rowan M.J., Selkoe D.J., Anwyl R. (2004). Block of long-term potentiation by naturally secreted and synthetic amyloid beta-peptide in hippocampal slices is mediated via activation of the kinases c-Jun N-terminal kinase, cyclin-dependent kinase 5, and p38 mitogen-activated protein kinase as well as metabotropic glutamate receptor type 5. J. Neurosci..

[bib0170] Whitcomb D.J., Hogg E.L., Regan P., Piers T., Narayan P., Whitehead G., Winters B.L., Kim D.H., Kim E., St George-Hyslop P. (2015). Intracellular oligomeric amyloid-beta rapidly regulates GluA1 subunit of AMPA receptor in the hippocampus. Sci. Rep-Uk..

[bib0175] Wilms C.D., Eilers J. (2007). Photo-physical properties of Ca2+-indicator dyes suitable for two-photon fluorescence-lifetime recordings. J. Microsci..

[bib0180] Wilms C.D., Schmidt H., Eilers J. (2006). Quantitative two-photon Ca2+ imaging via fluorescence lifetime analysis. Cell Calcium.

[bib0185] Witt M.R., Dekermendjian K., Frandsen A., Schousboe A., Nielsen M. (1994). 1994: Complex correlation between excitatory amino acid-induced increase in the intracellular Ca2+ concentration and subsequent loss of neuronal function in individual neocortical neurons in culture. Proc. Natl. Acad. Sci. U. S. A..

[bib0190] Zheng K., Bard L., Reynolds J.P., Jensen T.P., Gourine A.V., Rusakov D.A. (2015). Time-resolved imaging reveals heterogeneous landscapes of nanomolar Ca^2+^ in neurons and astroglia. Neuron.

